# Sulforaphane-cysteine-induced apoptosis via phosphorylated ERK1/2-mediated maspin pathway in human non-small cell lung cancer cells

**DOI:** 10.1038/cddiscovery.2017.25

**Published:** 2017-07-03

**Authors:** Kai Lin, Ronghui Yang, Zhongnan Zheng, Yan Zhou, Yang Geng, Yabin Hu, Sai Wu, Wei Wu

**Affiliations:** 1 Department of Biochemistry and Molecular Biology, School of Basic Medical Sciences, Capital Medical University, Beijing, China; 2 Institute of Brain Tumor, Beijing Institute for Brain Disorders, Capital Medical University, Beijing, China

## Abstract

Sulforaphane (SFN) was demonstrated to induce apoptosis in a variety of cancers via multiple mechanisms. However, owing to a short half-life in circulation, SFN was not used for clinical treatment yet. Interestingly, SFN analog, sulforaphane-cysteine (SFN-Cys) has a longer half-life in metabolism, and we previously demonstrated that SFN-Cys inhibited invasion in human prostate cancer cells. Here, we would investigate whether SFN-Cys induces apoptosis and find the underlying mechanisms in human non-small cell lung cancer (NSCLC) cells. Western blots were used to test the molecular linkages among extracellular signal-regulated kinases 1/2 (ERK1/2) and downstream signal molecules. Flow cytometry and fluorescence microscopy were used to detect cell death. Cell proliferation assay showed that SFN-Cys inhibited cell viability following a dose-dependent manner. Abnormal cell morphology was viewed after the cells were exposed to SFN-Cys. Flow cytometry showed that SFN-Cys induced cell apoptosis via a dose-dependent manner. Further, SFN-Cys triggered the activation of ERK1/2, which resulted in the upregulation of maspin, Bax, cleaved caspase-3 and downregulation of pro-caspase-3, Bcl-2, *α*-tubulin. Meanwhile, we demonstrated that recombinant caspase-3 cleaved *α*-tubulin in the lysate of cells, which were treated by SFN-Cys. These data indicated that SFN-Cys activated the ERK1/2-mediated mitochondria signaling pathway with maspin upregulation and *α*-tubulin downregulation leading to apoptosis. These findings will help to develop a novel therapeutic to target NSCLC cells.

## Introduction

Mortality of lung cancer worldwide increased yearly. The patients suffered from non-small cell lung carcinoma (NSCLC) had an extremely low 5-year survival rate.^1^ The commonly used therapeutics for NSCLC including surgery, chemotherapy and radiotherapy were not greatly successful because of low efficacy, side effects or drug resistance.^2^ Thus, the development of novel agents to treat NSCLC is not only interesting, but pressing as well.

Epidemiological studies showed that plant-derived drugs, sulforaphane (SFN) and its analogs, have powerful potential to prevent growth of cancer cells. SFN was first found in cruciferous vegetables such as broccoli, brussels sprouts and cabbages, then proved to induce apoptosis during tumorigenesis.^3^ SFN is metabolized in vivo to generate metabolites, such as SFN-glutathione, SFN-cysteine-glycine, SFN-cysteine (SFN-Cys), and SFN-*N*-acetylcysteine.^4,5^ SFN metabolites rather than SFN are the major forms of factors in circulation and tissues. SFN-Cys was found to inhibit histone deacetylase activation and have a higher plasma concentration and longer half-life, which might contribute to the inhibition of cancer growth.^6^ However, whether or not SFN-Cys induced apoptosis in human NSCLC cells and the underlying mechanisms were not clear.

The extracellular signal-regulated kinases 1/2 (ERK1/2) regulate many cellular functions, including proliferation and differentiation.^7^ ERK1/2 phosphorylation regulates the activation of downstream proteins and signal transduction by various extracellular stimuli. We found that transient (<15 min) ERK1/2 phosphorylation contributed to cancer proliferation,^8^ whereas sustained (>15 min) ERK1/2 phosphorylation resulted in growth inhibition and apoptosis.^9^ We found that SFN induced apoptosis by activating ERK1/2 in human glioblastoma cells, and we just reported that SFN-Cys inhibited invasion by persistent ERK1/2 phosphorylation in human prostate cancer cells. Hence, we proposed that SFN-Cys might induce apoptosis by activating ERK1/2 and further regulating the downstream signaling molecules in NSCLC cells.

Maspin has been characterized as a suppressor of growth and metastasis influencing many cellular events such as cell motility, adhesion and apoptosis in a variety of cancer cells.^10^ Phosphorylated ERK1/2 might contribute to upregulation of maspin in NSCLC cells. Therefore, it is likely that SFN-Cys might activate ERK1/2 regulating maspin and downstream signaling molecules leading to apoptosis in human NSCLC cells. Bax is a pro-apoptotic protein and Bcl-2 is an anti-apoptotic protein.^11^ The interaction between two regulators might result in the increased permeabilization of mitochondrial outer membrane and release of cytochrome c and Smac, subsequently initiating apoptosis.^12^ The ratio of Bax to Bcl-2 determines whether cytochrome c can be released and then the caspase family is activated.^13^ The investigation of activated ERK1/2-mediated expression of Bax, Bcl-2 and caspase-3 is necessary to explain the signaling transduction of apoptosis. Apoptotic caspases are sub-categorized initiator caspases, such as caspase-2, caspase-8, caspase-9 and caspase-10 and executioner caspases, such as caspase-3, caspase-6 and caspase-7.^14,15,16,17^ Once initiator caspases are activated, they produce a series of chain reactions, activating several other executioner caspases.^15^ Executioner caspases might degrade over 600 cellular components such as poly (ADP-ribose) polymerase (PARP) in order to induce the morphological changes for apoptosis.^18^ Therefore, we think that SFN-Cys might induce apoptosis through ERK1/2-mediated maspin upregulation, mitochondria death pathway via regulating Bax/Bcl-2 and caspase-3. Meanwhile, cell skeletal protein *α*-tubulin is also a substrate of caspase-3, and plays a key role in cell growth, migration and nutrient transfer.^16^ Microtubules are constituted by *α*-tubulin and *β*-tubulin.^19^ They maintain dynamic balance of microtubule via polymerization and depolymerization. Once *α*-tubulin is disturbed, the dynamic balance is broken and apoptosis occurs.^20^ Investigation of SFN-Cys-induced *α*-tubulin downregulation is beneficial to find new mechanisms to elucidate SFN-Cys-induced apoptosis.

In summary, here we will investigate and find some novel mechanisms involved in SFN-Cys-induced apoptosis in NSCLC cells. These studies will assist us to develop new anticancer drugs with lower toxicity and resistance, higher safety and efficiency to treat cancer patients.

## Results

### SFN-Cys inhibits cell proliferation in a dose-dependent manner

**Figure 1:**
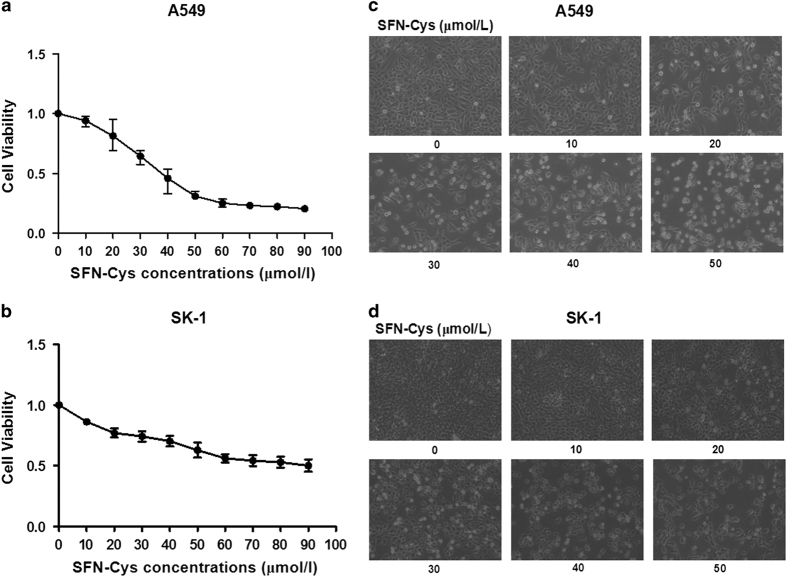
Cell viability analysis and morphological changes after treated with SFN-Cys. (**a**) A549 and (**b**) SK-1 cells were treated with various concentrations of SFN-Cys (0, 10, 20, 30, 40, 50, 60, 70 and 80 *μ*M) for 24 h. SFN-Cys significantly inhibited the proliferation of A549 and SK-1 cells in a dose-dependent manner. These results are presented as mean±S.D. from three independent experiments. Error bars indicate uncertainty errors in graphs to relay 95% of confidence in the interpreted data. The (**c**) A549 and (**d**) SK-1 cells were examined with Leica DMIRB Microscope at ×100 magnification. Cells treated with various concentrations of SFN-Cys (0, 10, 20, 30, 40 and 50 *μ*M) generated morphological changes.

Cells were treated with increasing concentrations of SFN-Cys (0, 10, 20, 30, 40, 50, 60, 70 and 80 *μ*M) for 24 h. The results showed that cell viability was decreased significantly after the cells were treated by SFN-Cys for 24 h in a dose-dependent manner (Figures 1a and b), indicating that SFN-Cys inhibited cell growth in those two cell types. In particular, the decreasing trends of cell viability in A549 cells are greater than those in SK-1 cells. More, the significant reduction of cell viability started at the dose of 20 *μ*M. Therefore, 20 *μ*M should be the optimal treatment concentration of SFN-Cys for the further studies.

### SFN-Cys changed cell morphology

Morphological observation showed that the cells treated with SFN-Cys were round and transparent with short pseudopodia (Figures 1c and d). With increasing concentrations, number of the dead cells increased. At the concentration of 20 *μ*M, there was an obvious cell death in A549 and SK-1 cells and simultaneously morphological changes were observed. These alterations of cell morphology might result from the inhibition of cell growth and apoptosis.

### SFN-Cys induced apoptosis in a dose-dependent manner

**Figure 2:**
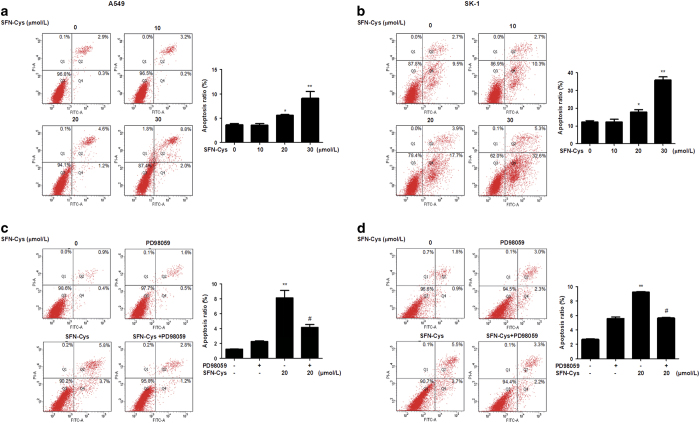
SFN-Cys-induced dose-dependent apoptosis in human A549 and SK-1 cells. (**a**) A549 and (**b**) SK-1 cells were treated with various concentrations of SFN-Cys (0, 10, 20 and 30 *μ*M) for 24 h, and stained with Annexin V-FITC/PI. Compared with the control cells, flow cytometry revealed that the apoptotic rates of the SFN-Cys treated (**c**) A549 and (**d**) SK-1 cells were significantly increased (*P*<0.01), whereas the apoptotic rates of the SFN-Cys-treated cells combined with PD98059 was declined (*P*<0.05). These results represented three independent tests. Error bars indicate uncertainty errors in graphs to relay 95% of confidence in the interpreted data. * Indicates *P*<0.05 *versus* the control group. ** Indicates *P*<0.01 *versus* control group. ^#^ Indicates *P*<0.05 *versus* the SFN-Cys-only group.

To confirm that SFN-Cys-induced inhibitory effect on NSCLC cells was due to cell apoptosis, flow cytometry assay was performed to verify and quantify the percentages of apoptotic cells. The percentages of the early and late apoptotic cells induced by SFN-Cys were represented in the lower right (LR) and upper right (UR) quadrant of the flow cytometry histograms, respectively. The total percentages of apoptotic A549 cells (UR+LR) increased in SFN-Cys-treated cells (10 *μ*M SFN-Cys, 3.4%; 20 *μ*M SFN-Cys, 5.8%; 30 *μ*M SFN-Cys, 10.8%), compared with non-treated cells (3.2%) for 24 h (Figure 2a). Similar data were observed in SK-1 cells, where the total percentages of apoptotic cells increased from 12.2% in control cells to 13.0%, 21.6% and 37.9% in cells treated with 10, 20 and 30 *μ*M SFN-Cys, respectively (Figure 2b). The results showed that treatment with 10, 20 and 30 *μ*M SFN-Cys for 24 h induced apoptosis in A549 and SK-1 cells in a dose-dependent manner. The results indicated that SFN-Cys exerted an anticancer effect on NSCLC cells.

Further, combined with PD98059, SFN-Cys-induced apoptosis was reversed in both cell types (Figures 2c and d). These data suggested that phosphorylated ERK1/2 contributed to SFN-Cys-induced apoptosis.

### SFN-Cys-induced apoptosis via sustained ERK1/2 phosphorylation

**Figure 3:**
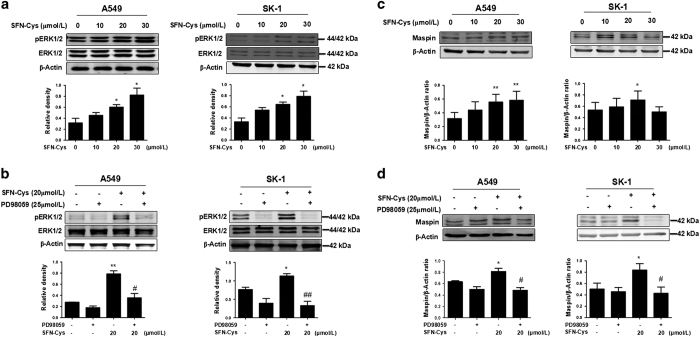
(**a**) Western blot analysis of the expression of ERK1/2 and pERK1/2 in A549 and SK-1 cells following treated with SFN-Cys (0, 10, 20 and 30 *μ*M) for 24 h. (**b**) A549 and SK-1 cells were cultured with 20 *μ*M SFN-Cys, 25 *μ*M PD98059, or a combination of the two compounds for 24 h, and the expression levels of ERK1/2 and pERK1/2 were analyzed. (**c**) Western blot was used to analyze the expression of maspin following treated with different concentrations of SFN-Cys (0, 10, 20 and 30 *μ*M). (**d**) The expression of maspin following treated with SFN-Cys and PD98059 alone or in combination. After treatment with PD98059 (25 *μ*M) for 30 min, the cells were incubated with 20 *μ*M SFN-Cys for 24 h. The upregulation of maspin induced by SFN-Cys was reversed by PD98059. *β*-Actin was used as a control. These results were represented as the mean±S.D. of three independent tests. Error bars indicate uncertainty errors in graphs to relay 95% of confidence in the interpreted data. *Indicates *P*<0.05 *versus* the control group. ** Indicates *P*<0.01 *versus* control group. ^#^ Indicates *P*<0.05 *versus* the SFN-Cys-only group. ^## ^Indicates *P*<0.01 *versus* SFN-Cys-only group.

Here we investigated whether the SFN-Cys-induced apoptosis of A549 and SK-1 cells was associated with the intracellular signaling pathways. Previous studies showed that phosphorylation of ERK1/2 reached the peak at 24 h.^21^ Therefore, we chose 24 h as the optimal time for subsequent study. The cells were treated with increasing doses of SFN-Cys (0, 10, 20 and 30 *μ*M) for 24 h. Western blot demonstrated that phosphorylation of ERK1/2 was significantly enhanced with the increased drug concentrations (Figure 3a). The results showed that SFN-Cys phosphorylated ERK1/2 via a sustained manner in both A549 and SK-1 cells. To examine whether PD98059 weakens SFN-Cys-induced inhibition in NSCLC cells, cells were cultured in the presence of either SFN-Cys (20 *μ*M) or PD98059 (25 *μ*M), respectively, or a combination of the two. The results revealed that the phosphorylation of ERK1/2 was significantly decreased in the cells treated with both SFN-Cys (20 *μ*M) and PD98059 (25 *μ*M) compared with the cells treated with SFN-Cys only (Figure 3b).

### SFN-Cys upregulated maspin via activating ERK1/2

Western blot analysis showed that maspin was upregulated in a dose-dependent manner (Figure 3c). Either SFN-Cys (20 *μ*M) or PD98059 (25 *μ*M), respectively, or a combination of the two were used to test the expression of maspin. Western blot showed that PD98059 diminished the upregulation of maspin (Figure 3d). These data indicated that activated ERK1/2 contributed to maspin upregulation.

### SFN-Cys upregulated Bax and downregulated Bcl-2 via sustained ERK1/2 phosphorylation

**Figure 4:**
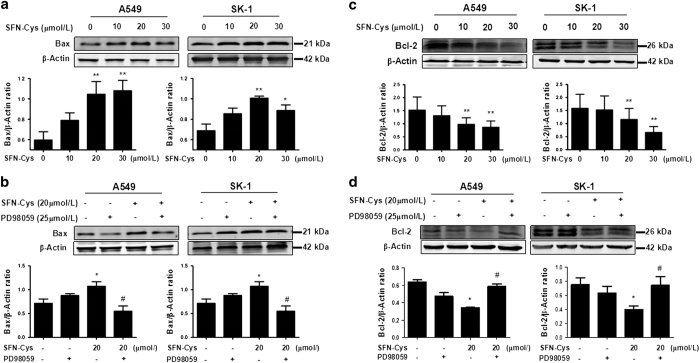
(**a**) SFN-Cys increased the expression of Bax by activating ERK1/2 and (**b**) the effect was reversed by PD98059. Western blot analysis was used to evaluate the levels of Bax. Furthermore, (**c**) SFN-Cys decreased the expression of Bcl-2 by activating ERK1/2 and (**d**) the effect was reversed by PD98059. Western blot was utilized to evaluate the levels of Bcl-2. Error bars indicate uncertainty errors in graphs to relay 95% of confidence in the interpreted data. * Indicates *P*<0.05 *versus* control group. ** Indicates *P*<0.01 *versus* control group. ^#^ Indicates *P*<0.05 *versus* SFN-Cys only group.

Western blot showed that SFN-Cys upregulated Bax expression (Figure 4a). The cells were treated with PD98059 to verify whether SFN-Cys regulated Bax via ERK1/2 phosphorylation. As a result, western blot showed that PD98059 was able to reverse the upregulation of Bax triggered by SFN-Cys (Figure 4b). These results indicated that SFN-Cys markedly upregulated Bax by phosphorylating ERK1/2. Inhibition of ERK1/2 activation through PD98059 blockade weakened the upregulation of Bax expression. Similarly, western blot showed that SFN-Cys downregulated Bcl-2 (Figure 4c). Furthermore, the cells were pretreated with PD98059 to verify whether this regulation of Bcl-2 resulted from the phosphorylation of ERK1/2. Significant changes were found between SFN-Cys-only and PD98059 plus SFN-Cys group, suggesting that blockade of ERK1/2 phosphorylation eradicated inhibition of Bcl-2 expression (Figure 4d). Thus, activated ERK1/2 might regulate Bax/Bcl-2 ratio leading to cell death in response to SFN-Cys.

### SFN-Cys-induced activation of caspase-3 and downregulated *α*-tubulin via activating ERK1/2

**Figure 5:**
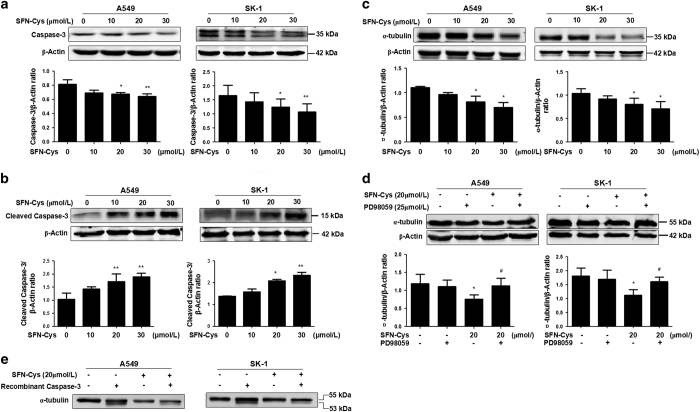
Western blot was utilized to evaluate the levels of pro-caspase-3, cleaved caspase-3 and *α*-tubulin. (**a**) SFN-Cys downregulated the level of pro-caspase-3. (**b**) SFN-Cys upregulated the expression of cleaved caspase-3 via increased concentrations; (**c**) and (**d**) SFN-Cys decreased *α*-tubulin, whereas PD98059 reserved this process. (**e**) *In vitro*, recombinant caspase-3 cleaved *α*-tubulin to produce a band of 53 kDa, suggesting that SFN-Cys activated caspase-3 might degrade *α*-tubulin. Each experiment was performed in triplicate. Error bars indicate uncertainty errors in graphs to relay 95% of confidence in the interpreted data. * Indicates *P*<0.05 *versus* control group. ** Indicates *P*<0.01 *versus* control group. ^#^ Indicates *P*<0.05 *versus* SFN-Cys only group.

**Figure 6:**
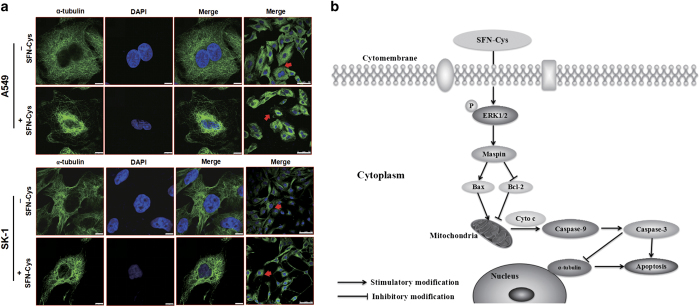
(**a**) The cells were treated with 20 *μ*M of SFN-Cys for 24 h and measured by laser scanning confocal microscopy. The cell nucleus was dyed purple and *α*-tubulin was colored green. Immunofluorescence showed that *α*-tubulin got fuzzy and loose arrangement in the treated cells compared with the normal cells. *α*-Tubulin expression was downregulated in response to SFN-Cys. All of the experimental values were determined via identical instrument settings to allow for a quantitative comparison of the cell-associated fluorescent intensity values. Scale bars, 7.5 and 50  *μ*m. (**b**) Assumed signaling pathway map for SFN-Cys-induced apoptosis in human NSCLC cells.

The cells were treated with increasing doses of SFN-Cys (0, 10, 20 and 30 *μ*M) for 24 h. Western blot showed that pro-caspase-3 was significantly decreased, whereas cleaved caspase-3 was significantly increased with the rising SFN-Cys concentrations in A549 and SK-1 cells (Figures 5a and b). In addition, we detected the expression of *α*-tubulin in the cells in response to SFN-Cys. Results showed that *α*-tubulin was significantly decreased with the increased drug concentrations (Figure 5c). Meanwhile, western blot showed that PD98059 reversed the downregulation of *α*-tubulin induced by SFN-Cys (Figure 5d). We also demonstrated that *α*-tubulin was cleaved by recombinant caspase-3 in the lysate of cells, which were treated with SFN-Cys in A549 and SK-1 cells (Figure 5e). We further detected the expression of *α*-tubulin in A549 and SK-1 cells by immunofluorescence. The results showed that SFN-Cys (20 *μ*M) treatment caused shorter cellular pseudopodia, and the level of *α*-tubulin was markedly reduced (Figure 6a). These results indicated that SFN-Cys might induce activation of caspase-3 leading to degradation of *α*-tubulin and apoptosis in A549 and SK-1 cells.

## Discussion

SFN is a potent chemopreventive agent inducing apoptosis in a variety of tumor cells.^22^ However, owing to the short half-life in circulation, SFN has not been used in the clinical treatment although a few clinical trials were done. However, SFN-Cys, as a major metabolite of SFN, was found to have extensive tissue distribution in the lung of treated mice and a longer half-life in circulation.^23^ We reported that SFN-Cys inhibited invasion in prostate cancer,^24^ but we do not know if it also inhibits cell growth in lung cancer cells. Here we evaluated its function and working mechanisms causing apoptosis in NSCLC cells. The results showed that SFN-Cys inhibited cell proliferation in A549 and SK-1 cells, which also provided an optimal concentration and treatment time to study apoptosis in vitro. Further, SFN-Cys phosphorylated ERK1/2 leading to apoptosis, which might result from the upregulation of maspin, Bax, cleaved caspase-3, and downregulation of Bcl-2, pro-caspase-3 and *α*-tubulin. Furthermore, recombinant caspase-3 cleaved *α*-tubulin in the cells treated with SFN-Cys. These findings revealed a novel mechanism, which SFN-Cys exhibited its pro-apoptotic effect in NSCLC cells (Figure 6b).

In the present model, we demonstrated that SFN-Cys inhibited cell proliferation and induced cell apoptosis in both A549 and SK-1 cells in a dose-dependent way. The cell viability showed a remarkable decrease and the apoptotic rates increased significantly following the treatment with 20 *μ*M SFN-Cys. The cell morphological changes with round shapes and shorter pseudopodia also indicated that SFN-Cys promoted apoptosis and inhibited invasion in human NSCLC cells. As a result of this, we further investigated the molecular mechanisms of SFN-Cys-mediated apoptosis. We found that SFN-Cys significantly increased ERK1/2 phosphorylation in a dose-dependent manner. However, the phosphorylated ERK1/2 inhibitor, PD98059 significantly reversed SFN-Cys-induced apoptosis. These indicated that SFN-Cys induced apoptosis through sustained ERK1/2 activation in human NSCLC cells.

ERK1/2 is the major kinase that plays a key role in numerous signaling pathways, and is often activated in cancer cells.^25^ Sustained activation of ERK1/2 contributed to apoptosis, and acute activation of ERK1/2 caused tumor initiation and progression.^26^ The ERK1/2 activation contributed to intracellular protein–protein interactions and the regulation of multiple cellular processes. Our previous studies demonstrated that SFN-Cys acted as a chemopreventive agent through activating ERK1/2 in human prostate cancer DU145 and PC3 cells, a few downstream effectors were involved.^24^ However, the linkage between ERK1/2 phosphorylation and the modulation of maspin on cell apoptosis has not been reported yet. Maspin is a tumor-suppressor protein, and its overexpression might induce apoptosis through regulating several members of Bcl-2 family.^27^ Interestingly, our results showed that SFN-Cys significantly increased the expression of maspin in NSCLC cells in a dose-dependent manner, suggesting that SFN-Cys-induced apoptosis might be associated with maspin upregulation. Particularly, once the SFN-Cys-treated cells were exposed to PD98059, the upregulation of maspin was reversed, suggesting that phosphorylated ERK1/2 upregulated maspin leading to apoptosis.

Maspin is the inducer of apoptosis via attaching to extracellular matrices, increasing sensitivity of apoptosis and inhibiting angiogenesis.^28^ Further, it is reported that the increased apoptosis in maspin-expressing cells lead to the changes in the protein levels of Bcl-2 family.^29^ Maspin linked to the upregulation of the pro-apoptotic protein Bax, which was translocated into the mitochondria during apoptosis.^30^ Bcl-2 is a member of the anti-apoptotic Bcl-2 family, which is involved in stabilizing mitochondrial membrane integrity.^31^ More studies have elaborated that Bcl-2 preserves the mitochondrial membrane and inhibits the release of internal calcium into the cytoplasm, whereas Bax is processed on the outer mitochondrial membrane and regulates the release of cytochrome c.^17,31^ The reasons are that Bcl-2 family proteins activated the intrinsic apoptotic pathway causing mitochondrial cytochrome c to be released into the cytosol.^12^ This molecule might bind an adaptor protein APAF-1, which recruits initiator caspase-9.^32^ Owing to the changed level of Bcl-2 family proteins, the apoptotic pathway became more active in maspin-expressing cells and this leads to the formation of a caspase activating multiprotein complex called the apoptosome, which accelerates apoptosis.^33^

Cell apoptosis is mainly induced by caspases, a family of cysteine aspartyl-specific proteases.^34^ Once activated, initiator caspases such as caspase-9 will cleave and activate other executioner caspases such as caspase-3 and caspase-7. This leads to degradation of cellular components for apoptosis.^17^ The caspase-3 and caspase-7 might in turn cleave a large amount of cellular substrates, such as PARP,^18^ which acts on mitochondria and activates cell death process through mitochondrial depolarization/membrane permeability transition and release of cytochrome c, apoptosis-inducing factor or endonuclease G into the cytosol.^35^ The previous studies showed that caspase-3 could cleave PARP to obtain the ability of detecting and repairing DNA damages, and then leading to cell apoptosis.^18^ Hence, we inferred that SFN-Cys-induced cell apoptosis might be involved in an intrinsic pathway.

In addition, we proved that caspase-3 cleaved *α*-tubulin, which is a vital component of cell microtubule. As a structural element of cell skeleton, microtubule supports cell structure and function stabilization, whereas mitotic spindles in eukaryotic cells segregate their chromosomes during cell division.^36^ Minor changes in microtubule might contribute to cell death; when *α*-tubulin was modified with tyrosination and acetylation, microtubules maintain a dynamic balance.^37^ Once the balance was destroyed, the microtubule structure broke up, apoptosis occurred.

In summary, our results revealed that SFN-Cys with longer half-life in circulation induced apoptosis in A549 and SK-1 cells via persistently activated ERK1/2, upregulating maspin and Bax, and downregulating Bcl-2 and *α*-tubulin, ultimately leading to the activation of caspase-3 in NSCLC cells. Thus, the present findings provided a new mechanism, which SFN-Cys induced apoptosis, suggesting that we might develop a novel therapeutic to treat human NSCLC.

## Materials and methods

### Reagents

SFN-Cys was the product of Santa Cruz Biotechnology (Santa Cruz, CA, USA). BCA protein assay kit was purchased from Invitrogen (Carlsbad, CA, USA). Annexin V-FITC apoptosis assay kit was purchased from GenStar (Beijing, China). Antibody against caspase-3 (1 : 200) and antibody against *β*-actin (1 : 5000) were purchased from ProteinTech Group, Inc. (Chicago, IL, USA). Anti-ERK1/2 (1 : 1000), anti-phospho-ERK1/2 (1 : 1000) and phosphorylated ERK1/2 inhibitor, PD98059 were purchased from Cell Signaling Technology, Inc. (Shanghai, China). Anti-maspin (1 : 1000), anti-Bax (1 : 1000), anti-*α*-tubulin (1 : 5000) and anti-Bcl-2 (1 : 500) were purchased from Sangon, Ltd (Shanghai, China). Recombinant caspase-3 was purchased from Sino Biological Inc. (Beijing, China). MTS assay kit was purchased from Promega Company (Madison, WI, USA).

### Cell culture

Two human NSCLC cell lines (A549 and SK-1) were purchased from the Cell Resource Center, Peking Union Medical College (CRC/PUMC, National Sciences & Technology Infrastructure (Beijing, China)). Cells were incubated in DMEM/F-12 culture medium with 10% FBS, 100 U/ml penicillin and streptomycin at 37 °C in a humidified incubator containing 5% CO_2_.

### Morphology assay

Cells at 70% confluence were exposed to SFN-Cys at a series of concentrations: 0, 10, 20, 30, 40 and 50 *μ*M for 24 h in a 6-cm dish. Cell morphology was viewed with phase contrast microscope at ×100 magnification (Leica, Wetzlar, Germany). Digital camera was connected to record cell morphology.

### Cell viability assay

Cell viability was evaluated via MTS assay kit as described in the specifications (Madison, WI, USA). Cells (4×10^3^) were seeded into a Corning Carbo-BIND 96-well plate and each well was added with a corresponding dose of SFN-Cys for 24 h. Next, 20 *μ*l of MTS reagent was added to each well and incubated at 37 °C for 1 h. The absorbance values were tested at 490 nm on a BioTek Synergy HT Multi-Detection Microplate Reader (BioTek, Winooski, VT, USA).

### Apoptosis assay

Apoptosis was determined by an Annexin V-fluorescein isothiocyanate (FITC)/propidium iodide (PI) assay. After the SFN-Cys treatment, cells were harvested and resuspended in 100 *μ*l binding buffer to reach a concentration of 1×10^6^ cells/ml. Further, 5 *μ*l Annexin V-FITC and 5 *μ*l PI (20 *μ*g/ml) were added to the cells, which were incubated for 15 min in the dark. A total of 400 *μ*l binding buffer was added to each tube, and the cells were tested via the BD LSRFortessa flow cytometer (Becton, Dickinson and Company, San Jose, CA, USA). These data were analyzed by WinMDI version 2.9 software (Purdue University Cytometry Laboratories, West Lafayette, IN, USA).

### Western blot

The treated cells were collected and lysed with lysis buffer (25 mM Tris-HCl, 150 mM NaCl, 1 mM EDTA, 1% NP-40, 5% glycerol, pH 7.4), the cell lysates were centrifuged at 12 500×*g* for 15 min. Equal amount of proteins were separated via 10% SDS-PAGE gels and transferred to nitrocellulose membranes. The membranes were blocked with 1.5% BSA for 1 h and subsequently incubated with the corresponding primary antibodies at 4 °C for 12 h (*α*-tubulin and *β*-actin were incubated at room tempreture for 30 min). After incubation, the membranes were washed with phosphate-buffered saline with Tween 20 (0.05%), and then incubated by the fluorescence-labeled secondary antibody (LI-COR Biosciences, Lincoln, NE, USA) for 1 h at room temperature. After washing, the protein bands were detected through the Odyssey Infrared Imaging System (LI-COR Biosciences). *β*-Actin was used as an internal control.

### Recombinant caspase-3 cleavage assay

A549 and SK-1 cells were incubated with 20 *μ*M SFN-Cys for 24 h and the collected cells were lysed in 25 mM Tris-HCl, 150 mM NaCl, 1 mM EDTA, 1% NP-40, 5% glycerol, pH 7.4 and 24 *μ*g protein was incubated with 10 *μ*l recombinant caspase-3 (Sino Biological Inc.) in 50 *μ*l reaction buffer containing 25 mM Hepes pH 7.5, 0.1% (w/v) Chaps, 10 mM DTT, at 37 °C for 6 h. After incubation, western blot analysis was used to detect *α*-tubulin.

### Immunofluorescence assay

The cells (4×10^4^) were incubated in a 24-well dish for 10 h first and then treated with 20 *μ*M SFN-Cys for 24 h. These cells were fixed with 4% paraformaldehyde for 20 min and permeabilized with 0.5% Triton X-100 for 15 min at room temperature. After blocking by 5% BSA for 30 min, the cells were incubated with primary antibodies (1 : 50) for 2 h and incubated with the fluorescence-labeled secondary antibody (1 : 500) for 1 h. The glass coverslips were stained with DAPI and the images were viewed on confocal laser scanning microscope (Olympus FV1000; Olympus Corp., Tokyo, Japan).

### Statistical analysis

Data are calculated as the mean±S.D. and statistical differences were analyzed by ANOVA followed by Student's *t*-test. All statistical analyses were done through SPSS 18.0 software package (International Business Machines Corporation, Armonk, NY, USA).
